# The Varicella-Zoster Virus Immediate-Early 63 protein affects chromatin controlled gene transcription in a cell-type dependent manner

**DOI:** 10.1186/1471-2199-8-99

**Published:** 2007-10-30

**Authors:** Lionel Habran, Nadia El Mjiyad, Emmanuel Di Valentin, Catherine Sadzot-Delvaux, Sébastien Bontems, Jacques Piette

**Affiliations:** 1 Virology & Immunology Unit, GIGA-Research, GIGA B34, University of Liège, B-4000 Liège, Belgium.

## Abstract

**Background:**

Varicella Zoster Virus Immediate Early 63 protein (IE63) has been shown to be essential for VZV replication, and critical for latency establishment. The activity of the protein as a transcriptional regulator is not fully clear yet. Using transient transfection assays, IE63 has been shown to repress viral and cellular promoters containing typical TATA boxes by interacting with general transcription factors.

**Results:**

In this paper, IE63 regulation properties on endogenous gene expression were evaluated using an oligonucleotide-based micro-array approach. We found that IE63 modulates the transcription of only a few genes in HeLa cells including genes implicated in transcription or immunity. Furthermore, we showed that this effect is mediated by a modification of RNA POL II binding on the promoters tested and that IE63 phosphorylation was essential for these effects. In MeWo cells, the number of genes whose transcription was modified by IE63 was somewhat higher, including genes implicated in signal transduction, transcription, immunity, and heat-shock signalling. While IE63 did not modify the basal expression of several NF-κB dependent genes such as IL-8, ICAM-1, and IκBα, it modulates transcription of these genes upon TNFα induction. This effect was obviously correlated with the amount of p65 binding to the promoter of these genes and with histone H3 acetylation and HDAC-3 removal.

**Conclusion:**

While IE63 only affected transcription of a small number of cellular genes, it interfered with the TNF-inducibility of several NF-κB dependent genes by the accelerated resynthesis of the inhibitor IκBα.

## Background

Varicella Zoster Virus (VZV) is the etiological agent of two clinically distinct diseases: varicella (chicken pox) as primary infection and zoster (shingles) after reactivation of latent virus from the dorsal root ganglia. Zoster is usually observed in elderly or immuno-compromised patients [1]. During latency, VZV does not express LAT as other α-Herpesvirus do, but viral proteins that are also present during lytic infection [2]. Transcripts from ORF4, 21, 29, 62, 63, and 66 [2-8], and several of the corresponding proteins have been detected in latently infected cells [8-12]. A recent study indicated that the expression of latency-related VZV genes, like ORF62 and 63, is regulated by epigenetic modifications of chromatin [13].

IE63 is of particular interest in VZV pathogenesis since it is abundantly expressed during acute infection and is the most abundant and most frequently identified of the six VZV gene products expressed during latency (reviewed in [14]). Its cellular localization is quite particular. Indeed, during a lytic infection it localizes mostly in the nucleus and slightly in the cytoplasm while during latency, it concentrates in the cytoplasm [8-10,15]. This protein is encoded by ORF63 and ORF70 and is the putative homologue of HSV ICP22. It has an apparent molecular mass of 45 kDa and is present in the virion tegument [16]. Moreover, IE63 interacts with VZV IE62 protein [5,17], is essential or not for VZV replication, depending on the cell-type studied [5,18,19] and critical for the establishment of latency [19]. In addition to regulatory functions, evidences were also provided that ORF63 promotes neuronal cell survival after VZV infection by modulating apopflsfto κis [20]. It has also been recently shown that expression of IE63 in the absence of other viral proteins blocked the antiviral effects of IFN-alpha by inhibiting phosphorylation of the alpha subunit of eukaryotic initiation factor 2 (eIF-2alpha) [21].

The activity of IE63 as a potential transcriptional regulator has been subject to controversy for years. It has been claimed that IE63 played only a minor role in the control of VZV gene expression [22]. However results from our laboratory showing that IE63 is able to down-regulate the expression of VZV immediate early (IE) and early (E) genes as well as heterologous viral and cellular promoters [23] suggest that IE63 has essential functions in the virus infectious cycle. Moreover, these repressive properties were shown to be dependent on the phosphorylation status of the protein [24,25]. Others found that IE63 could act as a co-stimulator of IE62 activity on the promoter of the VZV glycoprotein I (gI) [17]. It was alsoshown that IE63 could activate transcription driven by the cellular EF-1α promoter in the absence of other viral proteins in non-neuronal cells [26]. Finally, Desloges et al. added to the list of promoters regulated by IE63 one other heterologous promoter, the human GAPDH promoter [27]. Thus, based on currently published data, the effects of IE63 appear to be pleiotropic depending at least in part of the cell type and the promoter investigated.

The mechanisms by which IE63 could modulate gene transcription was poorly understood before it was shown that IE63 could mediate its repressive properties by disorganizing the pre-initiation complex by interacting with TFIIH, TFIIE, and RNA POL II [23]. In order to better understand the mechanism of action of IE63, we examined its effects on the regulation of cellular genes expression by full genome micro-array analysis in HeLa and MeWo cells infected with a lentivirus allowing the stable expression. We found that IE63 alone affected the transcription of a limited set of human genes. Furthermore, we showed that in HeLa cells the correct phosphorylation of IE63 is essential for the regulatory properties on endogenous promoters. Recently, a micro-array analysis of MRC5 cells infected with a recombinant adenovirus expressing IE63 has been published [28]. In this work, IE63 was shown to down-regulate the heat shock 70 kDa protein gene expression while several histone genes were found up-regulated.

In transient transfection assays, IE63 was demonstrated to down-regulate several cellular NF-κB-responding genes like IL-8 and IL-6 [23]. Surprisingly, these genes were not repressed in our experiments, as demonstrated by both micro-array analysis and real-time PCR. The fact that IE63 had no effect on basal expression of some NF-κB responsive genes may be due to a problem of promoter accessibility linked to chromatin opening. In order to assess this issue, we measured the expression rate of some of these genes (IL-8, IL-6, ICAM-1, and IκBα) in IE63 expressing HeLa cells after treatment with TNFα, a major pro-inflammatory cytokine known to increase chromatin accessibility of several promoters. We demonstrated that IE63 modulated IL-8, ICAM-1, and IκBα expression in response to TNFα via chromatin remodelling and modification of the recruitment of NF-κB p65 subunit.

## Results

### Varicella Zoster Virus IE63 protein affects transcription of a small number of genes in HeLa cells

**Figure 1:**
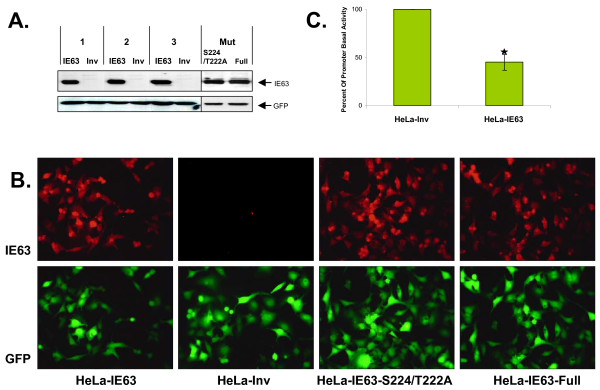
***VZV ORF 63 activity and expression by Lentivirus***. HeLa cells were infected with the lentivirus (Lenti-IE63, Lenti-Inv, Lenti-IE63-S224/T222A, Lenti-IE63-Full) in order to generate cell lines that stably express the protein IE63 wild-type (HeLa-IE63), in inverted orientation (HeLa-Inv) or mutated (HeLa-S224/T222A, HeLa-Full). One week after infection, cells were harvested. (A) Cells were lysed in radioimmunoprecipitation assay buffer and used for immunoblotting with mouse monoclonal antibody to the ORF63 protein or with rabbit polyclonal antibody to the EGFP. (B) Forty-eight hours post-seeding, immunostaining analysis was carried out using a monoclonal antibody (9A12) directed against IE63. Secondary antibody used is conjugated with Texas Red. (C) HeLa cells (HeLa-IE63, and HeLa-Inv) were transfected with 1 μg of pPol-Luc. 24 hours post-transfection, cells were harvested and the reporter gene activity was measured. Results are presented as a percentage of stimulation with respect to the basal expression of the promoter (= 100%). Data from luciferase assays were collected from six independent transfection experiments. ρ-values were calculated using the graphpad quickcalcs software [59]: *, significantly different from control (p-value < 0.05).

In order to study the influence of IE63 on human cellular transcription, three independent stable HeLa cell lines expressing IE63 protein were established. The whole genome expression profiles of HeLa cells infected with a recombinant lentivirus expressing VZV ORF63 (HeLa-IE63) and of HeLa cells infected with a recombinant lentivirus where IE63 gene was cloned in an inverted orientation (HeLa-Inv) were compared. In the lentivirus vectors used, the VZV gene was controlled by the EFIα promoter and was in frame with the EGPF gene but separated by an IRES region. All infections were carried out with the same multiplicity of infection. As expected, the three HeLa cell lines infected with the LentiIE63 (HeLa-IE63) expressed the EGFP protein (lower panel) and a protein of approximately 45 kDa recognized by a mouse monoclonal antibody directed against the VZV ORF63 protein (Fig.1A). IE63 was not detected in the three HeLa cell lines infected with the lentivirus where the ORF63 was cloned in the anti-sense direction (HeLa-Inv, control cells). Virtually 100% of HeLa cells expressed EGFP as determined by flow cytometry analysis (data not shown). Immunofluorescence studies were carried out to evaluate the proportion of HeLa cells expressing IE63 protein, and 100% of the cells expressing the EGFP protein also expressed IE63 (Fig. 1B).

**Figure 2:**
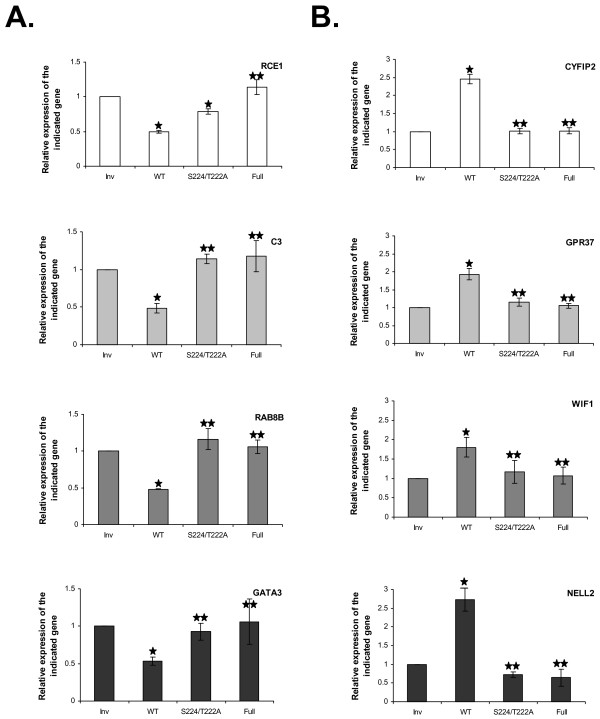
***Relative expression of selected cellular genes in HeLa-IE63, HeLa-IE63-S224/T222A, HeLai-IE63-Full versus control cells (HeLa-Inv)***. mRNA levels (A : RCE1, C3, RAB8B, and GATA3 ; B : CYFIP2, GPR37, WIF1, and NELL2) were determined by quantitative real-time PCR and normalized using the β2-microglobuline transcripts. Experiments were done at least in triplicate. Differences (n-fold) between samples were calculated using the standard-curve method and the 2^-ΔCt ^method. ρ-values were calculated using the graphpad quickcalcs software [59] : *, significantly different from control (Inv ; p-value < 0.05) ; **, not significantly different from control (Inv ; p-value ≥ 0.05).

Because the correct phosphorylation of IE63 was found essential for its repressive properties in transient transfection studies [24,25], HeLa cells stably expressing mutated forms of IE63 were generated : HeLa-IE63-S224/T222A, where two essential CDK1 phosphorylation residues (S224 and T222) were substituted by alanine [24], and HeLa-IE63-Full where all the phosphorylation target residues for CK1, CK2, and CDK1 were substituted by analaline [24]. The expression level and the proportion of cells expressing the mutated forms of IE63 protein were verified (Fig.1A, and 1B). The regulation properties of these mutated proteins on cellular genes were analyzed by real-time RT-PCR. As shown in Fig. 2, the abolition of phosphorylation drastically reduces the up- or down-regulating properties of IE63 on selected genes demonstrating that phosphorylation is essential for its activity on endogenous promoters.

To control whether IE63 was functional, HeLa cell lines expressing wild-type IE63 were transfected with a plasmid expressing a reporter gene (luciferase) under the control of the VZV DNA polymerase promoter which displayed a high basal activity and is known to be down-regulated by IE63 [25]. As shown in Fig. 1C, the basal activity of this promoter is down-regulated up to 50% in cells expressing IE63, demonstrating that IE63 is functional in these cells.

These cell lines expressing the wild-type IE63 protein were used for a micro-array analysis to delineate the effect of this viral transcription factor on cellular gene expression. For this, we used the U133A GeneChips (Affymetrix) representing 22,277 human genes probes set. Statistical filtering was done to include only genes with a modulation ρ-value < 0.05 (see materials and methods) and found similarly regulated in three independent experiments. As shown in Table 1, 17 genes were up-regulated and 9 were down-regulated in the three HeLa-IE63 cell lines relative to control cells (HeLa-Inv). Gene ontogeny analysis indicated that the predominant categories were signal transduction and transcription.

**Table 1:** Genes induced or repressed in HeLa-IE63 cells compared with control cells (Hela-Inv).

**Affimetrix designation**	**Entrez gene no.**	**Fold Induction**	**Gene Symbol**	**Description**
**Signal transduction**				
209631_s_at	2861	2.333	GPR37	G protein-coupled recept 37 (endoth recept type B-like)
204712_at	11197	2.192	WIF1	WNT inhibitory factor 1
210095_s_at	3486	1.911	IGFBP3	insulin-like growth factor binding protein 3
203474_at	10788	1.654	IQGAP2	IQ motif containing GTPase activating protein 2
209869_at	150	1.603	ADRA2A	Adrenergic, alpha-2A-, recept/adrenergic, alpha-2A-, recept
219210_s_at	51762	0.534	RAB8B	Member RAS oncogene family
**Transcription**				
213906_at	4603	1.677	MYBL1	v-myb myeloblastosis viral oncogene homolog (avian)-like 1
209604_s_at	2625	0.548	GATA3	GATA binding protein 3
215685_s_at	1746	0.507	DLX2	distal-less homeo box 2
**Immunity**				
217767_at	718	0.507	C3	complement component 3
Intracellular organelle				
212223_at	3423	1.7	IDS	Iduronate 2-sulfatase (Hunter syndrome)
203397_s_at	2591	1.579	GALNT3	UDP-N-acetyl-alpha-D-galactosamine(GalNAc-T3)
202843_at	4189	1.56	DNAJB9	DnaJ (Hsp40) homolog, subfamily B, member 9
205830_at	1047	1.648	CLGN	Calmegin
205333_s_at	9986	0.38	RCE1	RCE1 homolog, prenyl protein protease (S. cerevisiae)
211113_s_at	9619	0.588	ABCG1	ATP-binding cassette, sub-family G (WHITE), member 1
**Other/Unclassified**				
215785_s_at	26999	2.59	CYFIP2	Cytoplasmic FMR1 interacting protein 2
221901_at	85352	1.892	KIAA1644	KIAA1644 protein
208567_s_at	3768	1.889	KCNJ12	Potassium inwardly-rectifying channel, subfamily J, member 12
220393_at	51557	1.864	GLULD1	Glutamate-ammonia ligase domain containing 1
203413_at	4753	1.833	NELL2	NEL-like 2 (chicken)
215783_s_at	249	1.67	ALPL	Alkaline phosphatase, liver/bone/kidney
212158_at	6383	1.668	SDC2	Syndecan 2
213629_x_at	4494	0.582	MT1F	Metallothionein 1F
209946_at	7424	0.579	VEGFC	Vascular endothelial growth factor C
211066_x_at	134014	0.559	Protocad	Protocadherin gamma subfamily A, B and C

Real-time RT-PCR experiments were then carried out to confirm the results obtained from micro-array studies. RNA levels of 4 down-regulated (RCE1, C3, RAB8B, and GATA3; Fig. 2A) and four up-regulated (CYFIP2, GPR37, WIF1, and NELL2; Fig. 2B) genes measured by qRT-PCR confirmed the micro-array data.

### IE63 alters the recruitment of the Human RNA polymerase II on the WIF1 and C3 promoters

**Figure 3:**
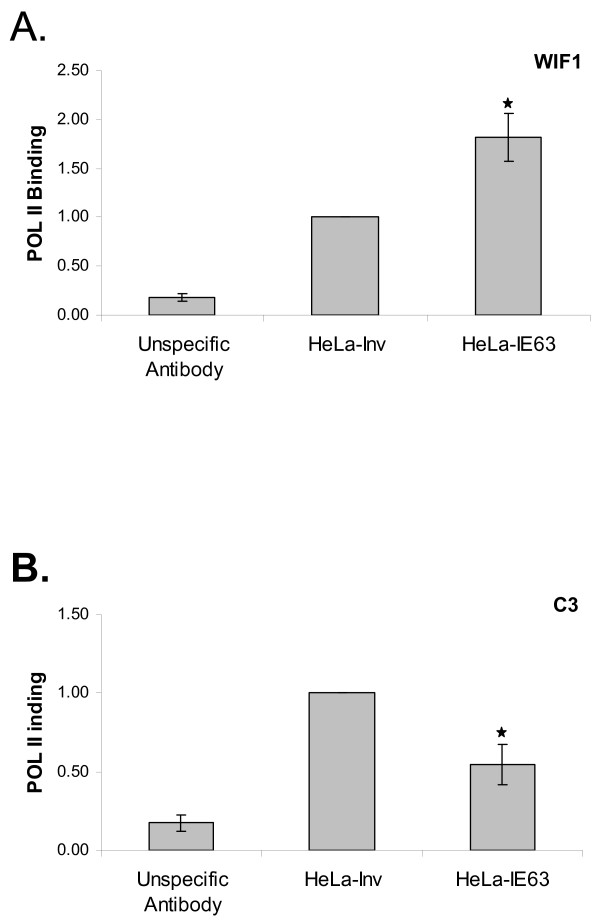
***RNA polymerase II recruitment on selected cellular genes in HeLa cells expressing or not IE63***. ChIP assay using RNA polymerase II antibody or unspecific antibody (lane 1) was performed on total cell lysates. Real-time PCR amplification of a 100 bp fragment from the WIF1 (A), and C3 (B) promoter encompassing [59] the transcription initiation site was carried out. ρ-values were calculated using the graphpad quickcalcs software [59]: *, significantly different from control (Inv ; p-value < 0.05).

IE63 was shown to be able to disrupt the transcriptional pre-initiation complex and to interact with several general transcription factors such as RNA POL II, TFIIH, and TFIIE [23]. In order to investigate whether IE63 diminished or enhanced the RNA POL II binding on endogenous human promoters, ChIP assays using antibody to RNA POL II were carried out on promoters either up- (WIF1) or down- (C3) regulated by IE63. As shown in Fig. 3, IE63 enhanced the RNA POL II binding on the WIF1 promoter up to 2-fold (Fig.3A), whereas RNA POL II binding was significantly decreased by IE63 on the C3 promoter (Fig.3B).

In conclusion, the results presented above showed that IE63 could modulate either positively or negatively the transcription on a restricted number of endogenous promoters and this effect required its proper phosphorylation by several cellular kinases.

### The effect of IE63 on human gene expression is cell-type dependent

**Figure 4:**
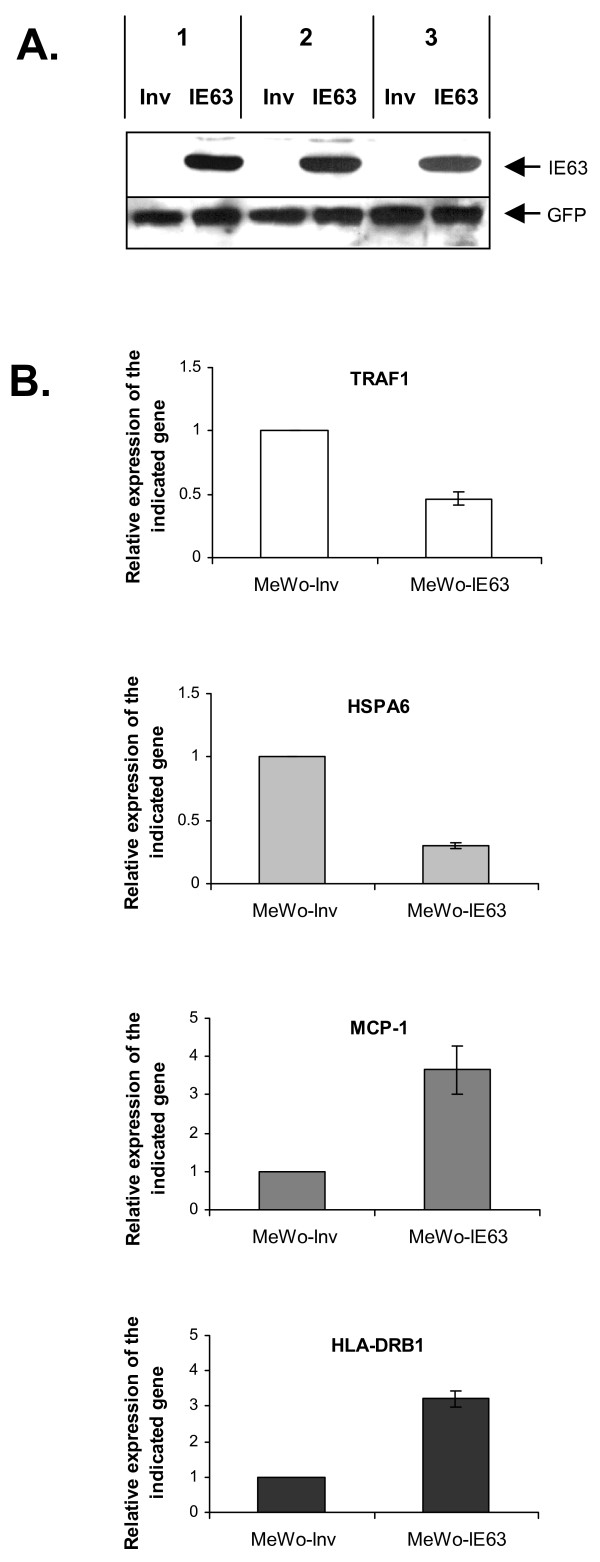
***Relative expression of selected cellular genes in MeWo-IE63 cells versus control cells***. **(A) **Cells were lysed in radioimmunoprecipitation assay buffer and used for immunoblotting with mouse monoclonal antibody to the ORF63 protein or with rabbit polyclonal antibody to the GFP. **(B) **mRNA levels (TRAF1, HSPA6, MCP-1, and HLA-DRB1) were determined by quantitative real-time PCR and normalized using the β2-microglobuline transcripts. Experiments were done at least in triplicate. Differences (n-fold) between samples were calculated using the standard-curve method and the 2^-ΔCt ^method. ρ-values were calculated using the graphpad quickcalcs software [59]: *, significantly different from control (Inv ; p-value < 0.05).

It has been recently shown that IE63 could modulate a different set of genes when expressed in MRC-5 cells [28]. We then decided to generate MeWo cells stably expressing IE63 in order to determine whether the modulation of gene expression is cell-type dependent. MeWo cells were then infected with the same lentivirus as above. As observed in HeLa cells, the proportion of cells expressing IE63 protein was almost 100% (data not shown). As shown in Fig. 4A, the expression level of IE63 is similar in the three independent MeWo cell lines generated. Micro-array analysis showed that the number of genes affected by IE63 was much higher in this cell-type than in HeLa cells (Table 2). Twenty-one genes were up-regulated and 75 genes were down-regulated more than 2-fold in MeWo-IE63 cells compared to control cells (Table 2). Gene ontogeny analysis indicated that the predominant categories were signal transduction, genes involved in transcription, which were all down-regulated, immunity/inflammation, chromatin, and 3 members of the heat shock protein 70 (HSP70) family. Real time RT-PCR experiments confirmed the results obtained with 2 up-regulated genes (HLA-DRB1, and MCP-1) and 2 down-regulated genes (TRAF1, and HSPA6) (Fig. 4B).

**Table 2:** Genes induced or repressed in MeWo-IE63 cells compared with control cells (MeWo-Inv).

**Affimetrix designation**	**Entrez gene no.**	**Fold Induction**	**Gene Symbol**	**Description**
**Signal Transduction**				
204425_at	393	0.50	**ARHGAP4**	Rho GTPase activating protein 4
204736_s_at	1464	0.50	**CSPG4**	chondroitin sulfate proteaoglycan 4
206028_s_at	10461	0.50	**MERTK **	c-mer proto-oncogene tyrosine kinase
206374_at	1850	0.50	**DUSP8**	dual specificity phosphatase 8
213221_s_at	23235	0.50	**KIAA0781**	KIAA0781 protein
206359_at	9021	0.38	**SSI-3 **	STAT induced STAT inhibitor 3
202328_s_at	5310	0.33	**PKD1**	polycystic kidney disease 1 (autosomal dominant)
**Transcription**				
201328_at	2114	0.50	**ETS2**	avian erythroblastosis virus E26 oncogene homolog 2
213668_s_at	6659	0.50	**SOX4 **	SRY (sex determining region Y)-box 4
213931_at	3398	0.50	**ID2**	inhibitor of DNA binding 2, dominant negative helix-loop-helix protein
215012_at	26036	0.47	**KIAA0576**	KIAA0576 protein
31637_s_at	732801	0.47	**Rev-ErbAalpha**	Rev-ErbAalpha
203873_at	6594	0.44	**SMARCA1**	Human global transcription activator homologous sequence mRNA
202672_s_at	467	0.41	**ATF3**	activating transcription factor 3
210426_x_at	6095	0.41	**RORA**	Human orphan hormone nuclear receptor RORalpha1 mRNA
202861_at	5187	0.38	**PER1**	Homo sapiens period (Drosophila) homolog 1
**Immunity/Inflamation**				
215193_x_at	3125	2.64	**HLA-DRB1**	MHC class II antigen
216598_s_at	6347	2.46	**MCP-1**	monocyte chemotactic protein
206569_at	11009	2.14	**ST16**	Homo sapiens suppression of tumorigenicity 16
211990_at	3113	2.14	**HLA-DPA1**	MHC class II DPw3-alpha-1 chain mRNA
208894_at	3122	2.00	**HLA-DRA**	MHC class II HLA-DR-alpha
209312_x_at	3123	2.00	**HLA-DRB1**	HLA-DRB (MHC class II antigen)
206026_s_at	7130	0.50	**TNFAIP6**	tumor necrosis factor, alpha-induced protein 6
209447_at	23345	0.50	**8B7**	lymphocyte membrane associated protein
214201_x_at	7916	0.47	**D6S51E**	HLA-B associated transcript-2
207850_at	2921	0.44	**GRO3**	GRO3 oncogene
217028_at	7852	0.38	**CXCR4**	receptor CXCR4
205599_at	7185	0.35	**TRAF1**	TNF receptor-associated factor 1
203665_at	3162	0.35	**HMOX1 **	heme oxygenase (decycling) 1
**Intracellular organelle**				
217452_s_at	8707	3.73	**B3GALT2**	2-acetamido-2-deoxy-D-glucose3beta- galactosyltransferase
205069_s_at	23092	2.83	**KIAA0621**	KIAA0621 protein
219250_s_at	23767	2.30	**FLRT3**	FLRT3 (fibronectin leucine rich transmembrane protein 3)
211205_x_at	8394	2.14	**PIP5K1A**	68 kDa type I phosphatidylinositol-4-phosphate5-kinase alpha
205547_s_at	6876	2.00	**TAGLN**	transgelin
204293_at	6448	0.50	**SGSH**	N-sulfoglucosamine sulfohydrolase
209893_s_at	2526	0.50	**FUT4**	ELAM-1 ligand fucosyltransferase
205830_at	1047	0.38	**CLGN**	Homo sapiens calmegin
207949_s_at	3382	0.33	**ICA1**	islet cell autoantigen 1 (69 kD)
214341_at	8906	0.02	**AP1G2**	adaptor-related protein complex 1, gamma 2 subunit
**Chromatin**				
212257_s_at	6595	2.00	**SMARCA2**	SWISNF related actin dependent regulator of chromatin, sf a, mb 2
210387_at	8339	2.00	**H2BFA**	H2B histone family, member A
**Heat-Shock**				
202581_at	3304	0.50	**HSPA1B**	heat shock 70 kD protein 1B
202843_at	4189	0.47	**MDG1**	microvascular endothelial differentiation gene 1
217911_s_at	9531	0.47	**BAG3**	BCL2-associated athanogene 3
117_at	3311	0.27	**HSP70B**	HSP70B Human heat-shock protein HSP70B gene
213418_at	3310	0.22	**HSPA6**	heat shock 70 kD protein 6 (HSP70B)
**Other/Unclassified**				
204730_at	9783	5.66	**KIAA0237**	KIAA0237 gene product
206021_at	54581	3.03	**SCAND2**	SCAN domain-containing 2
222108_at	347902	2.83	**AMIGO-2**	amphoterin-induced protein 2 precursor
218332_at	55859	2.64	**HBEX2**	hypothalamus protein
214079_at	10901	2.30	**SDR2**	dehydrogenase/reductase (SDR family) member 2
208282_x_at	57055	2.00	**DAZ2**	deleted in azoospermia 2
205960_at	5166	2.00	**PDK4**	Homo sapiens pyruvate dehydrogenase kinase, isoenzyme 4
211814_s_at	9134	2.00	**CCNE2**	cyclin E2 splice variant 1
202842_s_at	4189	0.50	**DKFZp564F1862**	hypothetical protein
222175_s_at	51586	0.50	**FLJ00003**	FLJ00003 protein
201500_s_at	6992	0.50	**PPP1R11**	PPP1R11
203045_at	4814	0.50	**NINJ1**	ninjurin 1
206307_s_at	2297	0.50	**FOXD1**	forkhead box D1
216594_x_at	1645	0.50	**chlordecone **	chlordecone reductase homolog
220058_at	79018	0.50	**MGC3048**	hypothetical protein MGC3048
221213_s_at	54816	0.50	**FLJ20086**	hypothetical protein FLJ20086
218145_at	57761	0.50	**LOC57761**	protein kinase domains containing proteinsimilar to phosphoprotein C8FW
219401_at	64132	0.50	**XT2 **	xylosyltransferase II
220587_s_at	64223	0.50	**GBL**	G protein beta subunit-like
214782_at	11016626	0.47	**FLJ13271**	cDNA FLJ13271 fis
214814_at	11684382	0.47	**DKFZp564P056**	cDNA DKFZp564P056
218651_s_at	55323	0.47	**FLJ11196**	hypothetical protein FLJ11196
219039_at	54910	0.47	**FLJ20369**	hypothetical protein FLJ20369
221836_s_at	6697854	0.47	**FLJ22741**	Homo sapiens cDNA: FLJ22741 fis
203431_s_at	9743	0.47	**KIAA0712**	KIAA0712 gene product
203504_s_at	19	0.47	**ABCA1**	ATP-binding cassette, sub-family A, member 1
209947_at	9898	0.47	**KIAA0144**	KIAA0144 gene product
218751_s_at	55294	0.44	**FLJ11071**	hypothetical protein FLJ11071
215078_at	4914612	0.44	**DKFZp564M2422**	cDNA DKFZp564M2422
204623_at	7033	0.44	**TFF3**	Homo sapiens trefoil factor 3 (intestinal)
209182_s_at	11067	0.44	**DEPP**	decidual protein induced by progesterone
215670_s_at	10434472	0.41	**FLJ12782**	cDNA FLJ12782 fis
218920_at	54540	0.41	**FLJ10404**	Homo sapiens hypothetical protein
219747_at	79625	0.41	**FLJ23191**	Homo sapiens hypothetical protein FLJ23191
204469_at	5803	0.41	**PTPRZ1**	protein tyrosine phosphatase, receptor-type, Z polypeptide 1
212531_at	3934	0.41	**LCN2**	Homo sapiens lipocalin 2 (oncogene 24p3)
211030_s_at	13623302	0.38	**MGC:10619**	clone MGC:10619
221959_at	10032854	0.38	**FLJ22488**	FLJ22488 fis
209160_at	8644	0.38	**c-hluPGFS**	Homo sapiens mRNA for hluPGFS
212839_s_at	6738	0.38	**SSA2**	Sjogren syndrome antigen A2 (60 kD, ribonucleoprotein autoantigen SS-ARo)
218864_at	7145	0.35	**PRO0929**	PRO0929 mRNA
220123_at	80255	0.35	**FLJ22004**	hypothetical protein FLJ22004
207992_s_at	272	0.35	**AMPD3**	adenosine monophosphate deaminase (isoform E)
213158_at	1523376	0.33	**DKFZp586B211**	cDNA DKFZp586B211
213445_at	23144	0.33	**KIAA0150**	KIAA0150 protein
219797_at	11320	0.33	**MGAT4A**	mannosyl-glycoprotein beta-acetylglucosaminyltransferase
219935_at	11096	0.31	**ADAMTS5**	disintegrin-like and metalloprotease with thrombospondin type 1 motif
213156_at	12761337	0.29	**DKFZp586B211**	cDNA DKFZp586B211
216006_at	24694	0.27	**24694**	clone 24694
206273_at	10650	0.27	**HFL-EDDG1**	erythroid differentiation and denucleation factor 1
218790_s_at	55217	0.19	**FLJ10727**	hypothetical protein FLJ10727
211037_s_at	13623420	0.07	**MGC:13124**	clone MGC:13124
210709_at	7770220	0.06	**PRO2710**	PRO2710 mRNA
209613_s_at	125	0.02	**ADH2**	alcohol dehydrogenase beta-1 subunit
217177_s_at	5262528	0.01	**FLJ13658**	cDNA DKFZp564N2216

### IE63 modulates gene transcription induced by TNFα

**Figure 5:**
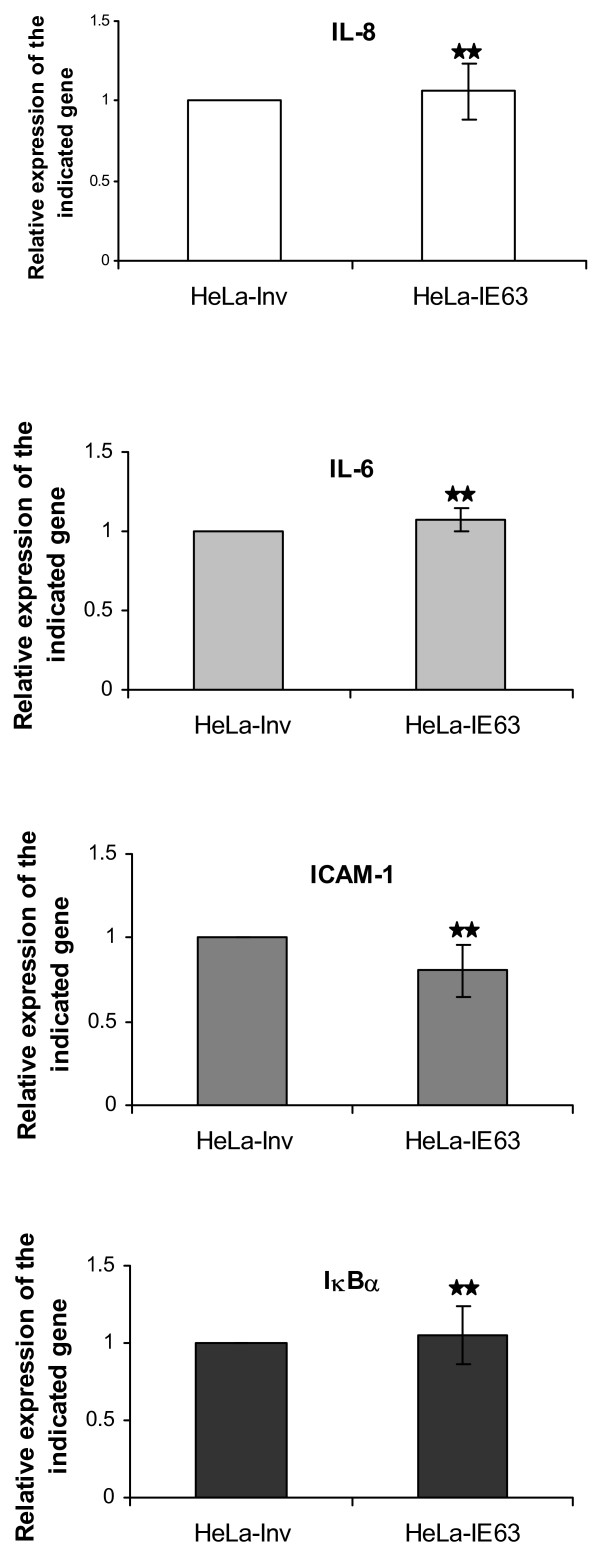
***Relative basal expression of IL-8, IL-6, ICAM-1 and IκBα genes in HeLa-IE63 cells compared to control cells***. mRNA levels (IL-8, IL-6, ICAM-1, and IκBα) were determined by quantitative real-time PCR in unstimulated HeLa cells expressing or not IE63 and normalized using the β2-microglobuline transcripts. Experiments were done at least in triplicate. Differences (n-fold) between samples were calculated using the standard-curve method and the 2^-ΔCt ^method. ρ-values were calculated using the graphpad quickcalcs software [59] : **, not significantly different from control (Inv ; p-value ≥ 0.05).

**Figure 6:**
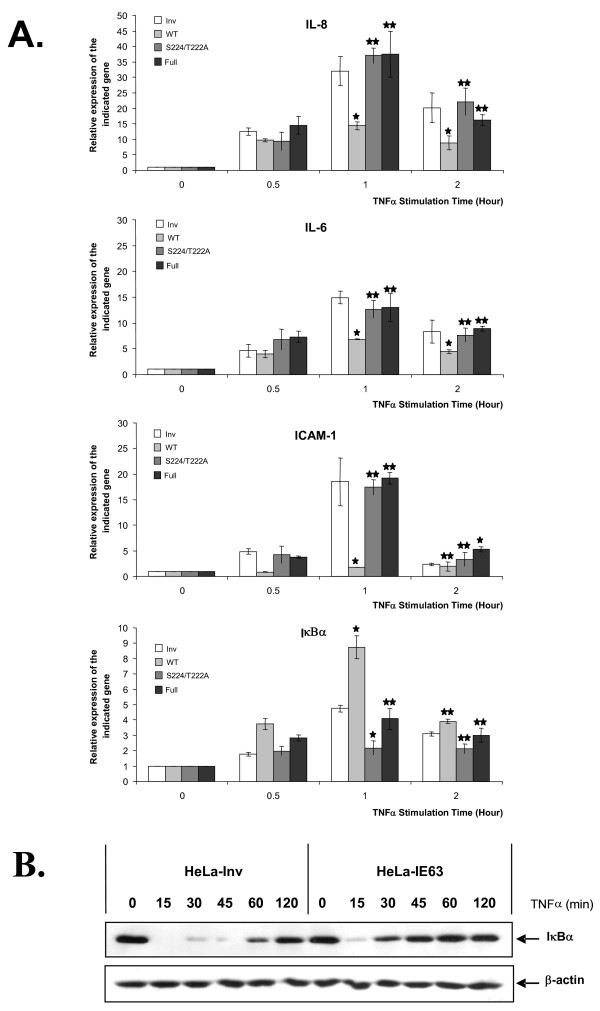
***Relative expression of IL-8, IL-6, ICAM-1 and IκBα genes in HeLa cells expressing IE63, IE63-S224/T222A, and IE63-Full versus control cells stimulated or not with TNFα***. HeLa cells expressing IE63 wild-type, IE63-S224/T222A, IE63-Full or IE63 in inverted orientation (Inv, control) were treated for increasing times (from 0 to 2 h) with TNFα at a final concentration of 200 U/mL. (A) Total RNA extracts were isolated and analyzed by Real-time RT-PCR using primers for the IL-8 mRNA, IL-6 mRNA, ICAM-1 mRNA, and IκBα mRNA. (B) The IκBα degradation. HeLa cells expressing IE63wt or control cells were treated for increasing times (from 0 to 2 h) with TNFα (200 U/mL). IκBα degradation was followed by Western Blotting on total cellular extracts. β-actin Western Blotting detection was used as loading control (lower panel). ρ-values were calculated using the graphpad quickcalcs software [59]: *, significantly different from control (Inv ; p-value < 0.05) ; **, not significantly different from control (Inv ; p-value ≥ 0.05).

Previous works from our laboratory have shown that transiently expressed IE63 is able to down-regulate several cellular NF-κB-responding genes like IL-8 and IL-6 [23]. Surprisingly, the basal expression level of these genes was not repressed in our experiments, as demonstrated by both micro-array analysis (Table 1 and 2) and real-time RT-PCR (Fig. 5). We also investigated the expression pattern of two other NF-κB-responding genes, ICAM-1 and IκBα, and we showed that the basal expression of these genes was not altered by IE63. The fact that IE63 had no effect on basal expression of some NF-κB responsive genes may be due to a problem of promoter accessibility linked to chromatin. In order to assess this issue, we measured the transcription level of these genes (IL-8, IL-6, ICAM-1, and IκBα) in IE63 expressing HeLa cells after treatment with TNFα, a cytokine known to increase chromatin accessibility of several genes. Once HeLa cells were stimulated by TNFα for increasing times (from 0 to 2 h), it turned out that the presence of IE63 reduced the TNFα-mediated expression of IL-8, IL-6, and ICAM-1 (Fig. 6A). Surprisingly, the transcription of IκBα gene was selectively increased by the presence of IE63 (Fig.6A). This unexpected result was confirmed by Western blot analysis. For this, HeLa cells expressing or not IE63 were treated for increasing times (from 0 to 2 h) with TNFα at a final concentration of 200 U/mL. As shown in Fig. 6B, in control HeLa cells a complete IκBα degradation could be observed after 15 min before observing its resynthesis after 60 min. In IE63 expressing HeLa cells, a complete IκBα degradation could also be observed 15 min after TNFα addition, but its resynthesis could be observed as soon as 30 min demonstrating that the presence of IE63 accelerated the replenishment of the IκBα pool.

The phosphorylation status of IE63 was shown to be crucial for influencing the TNFα-mediated gene transcription. For this, we compared IL-8, IL-6, ICAM-1, and IκBα RNA levels in HeLa cells expressing either IE63, or the two mutated forms (IE63-S224/T222A, and IE63-Full). As it shown in Fig. 6A, when phosphorylation is suppressed by alanine substitution, IE63 did not significantly modulate the TNFα-induced gene transcription, demonstrating the importance of correct phosphorylation of IE63 for this property.

### IE63 perturbs chromatin structure and NF-κB binding on the TNFα modulated gene promoters

**Figure 7:**
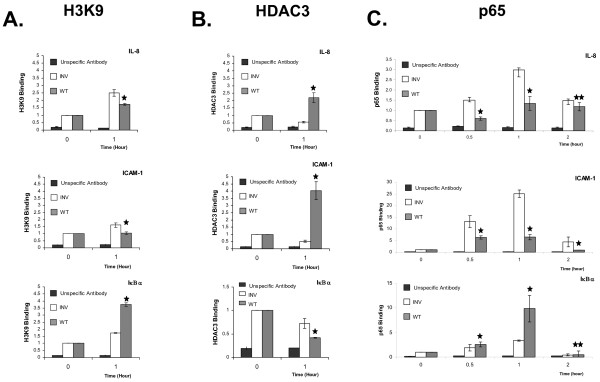
***HDAC3, NF-κB recruitment, and Histone H3 acetylation on selected cellular genes in HeLa cells expressing IE63 versus control cells***. (A, and B) HeLa cells were treated for one hour with TNFα (200 U/mL) and ChIP assay analysis was performed using an antibody specific of the K9 acetylated form of the histone H3 (A) and the histone deacetylase HDAC3 (B). An unspecific antibody (Flag) was used for evaluating specificity. (C) HeLa cells were treated for increasing times (from 0 to 2 h) with TNFα at a final concentration of 200 U/mL. ChIP assay using p65 antibodies was performed on total cell lysates. Real-time PCR amplification of a 100 bp fragment from the IL-8, ICAM-1 and IκBα promoter encompassing the proximal NF-κB site was carried out. ρ-values were calculated using the graphpad quickcalcs software [59] : *, significantly different from control (Inv ; p-value < 0.05) ; **, not significantly different from control (Inv ; p-value ≥ 0.05).

To go one step further in the understanding of the mechanism by which IE63 modified IL-8, ICAM-1, and IκBα inducibility by TNFα, chromatin immuno-precipitation assays (ChIP) were carried out. Histone acetylation and histone deacetylase (HDAC) removal were often associated with an increased chromatin accessibility at NF-κB-responsive promoters [29]. Using an antibody directed against the acetylated lysine 9 of histone H3 in ChIP assay, it was obvious that IE63 expression led to opposite effects: it reduced IL-8 and ICAM-1 promoters accessibility whereas it increased IκBα promoter accessibility upon TNFα (Fig.7A). The decreased accessibility of the IL-8 and ICAM-1 promoters mediated by IE63 correlated very well with a recruitment of HDAC3 while the reverse was observed with IκBα promoter (Fig. 7B).

Since results from our laboratory (El Mjiyad et al., submitted) showed that VZV is able to interfere with the immune response by inhibiting NF-κB binding to specific promoters, we investigated the *in vivo *recruitment of the NF-κB subunit p65 on IL-8, ICAM-1, and IκBα promoters. HeLa-IE63 and control cells (HeLa-Inv) were treated during increasing times (from 0 to 2 h) with TNFα (200 U/mL). After chromatin immunoprecipitation using specific antibodies to p65, quantitative PCR amplification of a region of 100 bp surrounding the NF-κB site(s) on the IL-8, ICAM-1 and IκBα promoters was carried out. As shown in Fig. 7C, the cells treatment during one hour with TNFα induced a 3- and 25-fold increase of p65 binding at the NF-κB proximal site of the IL-8, and ICAM-1 promoter, respectively. In HeLa-IE63 cells, this increase was significantly diminished. We then looked at IκBα promoter, and it turned out that the presence of IE63 increased p65 binding (Fig. 7C). Indeed, after one hour of TNFα treatment, a 3-fold increase of p65 binding was observed in control cells, whereas in IE63 expressing HeLa cells, a 10-fold increase was observable, demonstrating again that IE63 enhanced p65 recruitment at the IκBα promoter.

## Discussion

IE63 is of particular interest in Varicella Zoster Virus pathogenesis since it is abundantly expressed during acute as well as latent infection [15]. The activity of IE63 as a potential transcriptional regulator has been subject to controversy for years, being described either as an activator or a repressor of transcription [17,22-27].

To clarify this issue, the first part of this work was devoted to the examination of the effects of IE63 on cellular gene expression using a whole genome micro-array analysis in HeLa and MeWo cells stably expressing IE63. The main results obtained in this study can be summarized as follow: (i) IE63 alone affected on a cell-type mode the transcription of a limited number of human genes, including genes implicated in signal transduction, transcription, immunity, and heat-shock signalling. (ii) IE63 expression led to an inhibition or an increase of RNA POL II binding on the down- and up-regulated promoters tested here. (iii) In HeLa cells, the correct phosphorylation of the protein is a crucial event for its regulatory properties on endogenous promoters.

Expression of IE63 protein by a lentivirus influenced the transcription of relatively few cellular genes in HeLa (26 genes) and MeWo cells (106 genes). Previous micro-array studies performed with VZV-infected cells indicated that VZV was able to modify the expression of a variable number of genes depending on the cell type used [30,31]. Comparative micro-array analysis of IE63 expressing cells demonstrated many differences in depending on the cell type considered. Indeed, Hoover *et al. *[28] showed that, in human diploid fibroblasts (MRC-5), 71 genes of approximately 33,000 genes were up-regulated and 23 were down-regulated by IE63 protein. In human neuroblastoma (SKNSH), no significant differences in the expression level of cellular genes was found between control and IE63 expressing cells [28].

Several explanations might be proposed to explain these differences in response to IE63. First, as we and other have already shown, IE63 is a heavily phosphorylated protein and the phosphorylation pattern is cell-type dependent [17,24,25,32]. Another reason may be found in the chromatin context and promoter accessibility that may be different in the two cell types investigated. In the various works cited above, IE63 was expressed in several cellular backgrounds whose gene expression is likely to be differently affected by growth in cell culture. A last explanation is the viral vector used. Hoover *et al. *measured the effect of IE63 as soon as two days after cell infection with an adenovirus [28] whereas lentivirus was used in our work. It could be conceivable that infection mediated by adenovirus increased by itself chromatin accessibility allowing IE63 to access to a greater number of promoters, and then having a higher influence on cellular genes expression.

It must also be pointed out that the comparison between IE63 expression in MRC5 [28] and MeWo cells exhibited several common features. Histone H2 coding genes were up-regulated in both cell types while Heat Shock Protein 70 (hsp70) and TNFα-induced protein (TNFAIP) genes were down-regulated. While several viruses (including Herpesvirus and Adenovirus) have been reported to induce hsp70 genes [33-36], it is not clear yet why IE63 specifically and drastically down-regulated the members of this family.

Among the rather limited list of genes regulated in HeLa cells mediated by IE63, the down-regulation of the gene encoding the component protein 3 could be noticed. The complement system comprises several dozen proteins circulating in serum, or attached to cell surfaces, and orchestrating three distinct cascades (classical, alternative, and lectin pathways) into antimicrobial effector activities that range from the opsonization of foreign particles, the recruitment of phagocytes, to the lysis of infected cells [37]. The complement cascade is under tight cellular control by host inhibitor proteins, and it is perhaps not surprising that VZV has an inhibitory effect as an anti-complement defence system. Some viruses, for example HCMV, induce the expression of cellular complement inhibitors like DAF at the surface of infected cells [38]. The Epstein-Barr virus (EBV) also has complement regulatory activity against C3 [39]. Different studies have shown that HSV-1 and -2 gC proteins offer protection against viral neutralization mediated by complement [40,41].

When gene expression profiles obtained in this paper are compared to micro-array data obtained by the analysis of VZV infected cells [30,31], there are several global similarities that showed up, and among them, genes implicated in immunity/inflammation, signal transduction, and transcription in T cells, or in fibroblasts, as well as in VZV-infected skin xenografts. These classes of genes are also influenced when IE63 is expressed alone. For instance, among genes implicated in immunity/inflammation, TRAF (TNF Receptor-Associated Factor) genes are down-regulated in VZV-infected T cells as well as in IE63 expressing MeWo cells. Concerning signal transduction, two Ras oncogene family members and genes coding for G protein receptors were also down-regulated in both cases. Our work demonstrated that MCP-1 gene expression was stimulated by IE63, and this result is in accordance with what was shown by Jones *et al. *in VZV-infected cells [31].

Another important finding of this work was the demonstration that IE63 can differentially affect the TNF-inducibility of NF-κB regulated genes. The relevant results obtained here are: (i) in TNFα-stimulated cells, the expression of IL-8, IL-6, ICAM-1, and IκBα genes was affected by IE63 in a promoter-dependent manner, (ii) the phosphorylation status of the protein influenced these regulatory properties, (iii) the effects of IE63 were correlated with a modification of chromatin structure, and finally (iv) IE63 was able to modulate NF-κB binding on the tested promoters.

We have been able to show that TNFα treatment increases chromatin accessibility in the proximal area of the tested promoters as demonstrated by histone H3 acetylation and the inhibition of HDAC3 binding. Furthermore, the presence of IE63 modified chromatin in a different extent depending on the considered promoter. Unexpectedly, IE63 led to inhibition of chromatin accessibility mediated by TNFα on IL-8 and ICAM-1 genes, and to an increased accessibility in the context of the IκBα promoter. The inhibition of IL-8, and ICAM-1 gene expression could very well be a consequence of the induction of IκBα, since this protein is an inhibitor of NF-κB [42,43].

NF-κB plays a central role in the innate and adaptive immune response. The activation of this factor during early stages of viral infection leads to the expression of several immune response genes; such as pro-inflammatory cytokines (IFN-β, TNF-α, IL-6, IL-8), chemokines (RANTES) and adhesion molecules (ICAM-1, VCAM-1). NF-κB also strongly induces MHC-I and CD80/86 expression on antigen presenting cells, thus increasing T-cell activation [44]. NF-κB activation appears to be necessary for optimal replication of HSV-1 and HCMV early in infection, since it has been shown that blocking the NF-κB pathway decreased viral yields and inhibited the activation of several viral genes harbouring NF-κB response elements in their promoter [45,46]. A model of this interdependence between HCMV and NF-κB has been generated by Montag *et al. *[47]. In contrast, the lack of functional NF-κB response elements in the promoters of VZV IE genes allows suspecting that VZV replication cycle could be NF-κB independent. VZV may need to directly interfere with the NF-κB signalling in order to avoid downstream effects of NF-κB activation. Likely, NF-κB inhibition properties of VZV could at least in part be mediated by IE63. Such a modulation mechanism of NF-κB has already been demonstrated with HCMV. Indeed, Taylor *et al. *have shown that HCMV Immediate Early protein IE86 can suppress virus-induced pro-inflammatory cytokines transcripts expression by efficiently blocking the binding of NF-κB to cellular NF-κB responding genes [48]. Surprisingly it has been shown that VZV specifically induced interleukin-6 in human monocytes via TLR2-dependent activation of NF-κB [49]. Moreover Rahaus *et al*. have demonstrated that infection by VZV caused a significant increase in activation of JNK/SAPK in the early phase of infection and an increase in activation of p38/MAPK in the later phase. Subsequent cascades to induce pro-inflammatory responses were activated whereas cascades to activate apoptotic events were not [50]. This suggests that activation of stress pathways by VZV infection represents a finely regulated system that activates cellular transcription factors for transregulation of VZV-encoded genes, but prevents activation of cellular defence mechanisms [50].

The observation that IκBα transcription was stimulated when IE63 expressing cells were treated with TNFα suggests that the strategy by which VZV, may be via IE63, inhibits the NF-κB pathway may protect VZV-infected cells from antiviral responses induced by exogenous pro-inflammatory cytokines, many of which act through NF-κB signalling pathways.

One of the main information brought by this work is the capacity of the IE63 to exert epigenetic effects on NF-κB regulated promoters. Similar effects on chromatin have been observed with other α-Herpsvirus. Indeed, in HSV-1 infected cells, ICP0, the homologue of VZV ORF61 protein, has been shown to block viral genes silencing by interacting with HDAC1 [51]. Moreover, the U_S_3 kinase (the VZV ORF66 kinase homolog) can also block this silencing in a mechanism distinct from that of ICP0 [52,53]. It has been demonstrated that during lytic infection HSV-1 is associated with histone bearing modifications that correlate with active transcription [51], ICP0 may be responsible for this chromatin permissiveness. Concerning the Bovine Herpesvirus 1 (BHV-1), it has been shown that bICP0 can associate with HDAC1 to relieve HDAC1-mediated repression of viral gene transcription [54]. Furthermore, this protein was able to interact with p300/CBP (CREB Binding Protein) and this interaction cooperate to enhance viral gene expression and may interfere with antiviral signalling [55].

## Conclusion

In conclusion, this work clearly demonstrated that stable expression of IE63 in two cellular backgrounds only affected the basal expression of a rather limited number of genes while it differentially modulated the TNFα-inducibility of several NF-κB-regulated genes. While the molecular mechanisms governing these effects are not established yet, it can be suspected that IE63 is a major player in the complex interplay between VZV and the host immune system.

## Methods

### Cells lines

MeWo cells, a continuous human melanoma cell line (ECACC93082609), were grown in EMEM (Biowhittaker) supplemented with 10% foetal bovine serum (FBS, biowhittaker), 1% L-glutamine, and 1% non-essential amino acids (NEAA, GibcoBRL).

HeLa cells, human epithelial cells from a fatal cervical carcinoma transformed by human papillomavirus 18 (ATCC-CCL-2), were cultivated in Eagle's Modified Essential Medium (EMEM, Biowhittaker) supplemented with 10% foetal calf serum (FCS, Biowhittaker), and 1% L-glutamine.

293FT cells, derived from 293 cell line established from primary embryonic human kidney transformed with sheared human adenovirus type 5 DNA, were grown in Dulbecco's Modified Eagle's Medium (DMEM, Biowhittaker) supplemented with 10% FBS, 1% L-glutamine, 1% NEAA, and 1% Penicillin/Streptavidine (P/S).

Lentiviruses in which the ORF63 has been cloned in the sense (Lenti-IE63), or in the anti-sense orientation (Lenti-Inv, control) were produced by transient calcium phosphate co-transfection of 293FT cells with the plasmid carrying the IE63 gene (pTRIP-EFIα-ORF63-IRES-EGFP [56]), pCMV8.91 (a kind gift from Dr. D. Trono [57]) and pVSV-G (a kind gift from Dr. T. Friedmann [58]) as previously described [56]. A similar procedure was followed to generate a lentivirus in which the ORF63 was mutated either at S224/T222A (Lenti-IE63-S224/T222A), or on all the target residues of CK1, CK2, and CDK1, i.e. S15A, S150A, S157A, S165A, T171A, S173A, S181A, S185A, S186A, S197A, S200A, T201A, S203A, T222A, S224A, and T224A (Lenti-IE63-Full) [24]. All of these constructs contain the gene encoding the enhanced green fluorescent protein (EGFP). Lentivirus concentrations were determined by measuring the HIV-1 p24 capsid protein. The viral titers were measured on the CEMx174 cells and expressed in TU/mL (Transduction Unit/mL).

HeLa and MeWo cells were plated at 5 × 10^5 ^cells/mL and the lentiviral particles were added at the concentration of 500 ng/mL of viral p24. After 12h, cells were washed and cultured for 72 h. EGFP expression was confirmed by flow cytometry analysis. This procedure allowed us to generate four HeLa cell lines, i.e. HeLa-IE63, HeLa control cells (HeLa-Inv), HeLa-IE63-S224/T222A, and HeLa-IE63-Full, and two MeWo cell lines, MeWo-IE63, and control cells (MeWo-Inv).

### Antibodies and reagents

TNFα was purchased from Roche and used at a final concentration of 200 U/mL. The antibodies used in this work (Western blot analysis and immunofluorescence) are directed against IE63 (produced by Dr. S. Bontems, University of Liège, Belgium), Green Fluorescent Protein (GFP) (Rabbit polyclonal, 1/1000 ; Tebu Bio SC-8334), p65 (Rabbit polyclonal, 1/1000 ; Tebu Bio SC-372), IκBα (Mouse monoclonal, 1/400 ; kind gift from Ron Hay, Scotland), β-Actin (Mouse monoclonal, 1/1000 ; Sigma), H3AcK9 (Mouse monoclonal, 1/1000 ; Upstate Cell Signaling), HDAC3 (Mouse monoclonal, 1/1000 ; Upstate Cell Signaling), Histone H3 (Mouse monoclonal, 1/500 ; Abcam), and Human RNA polymerase II (Rabbit polyclonal, 1/200 ; Tebu Bio).

### Immunofluorescence

HeLa and MeWo control cells or expressing wild-type or mutated IE63 were seeded on coverslides into 10-mm dishes and grown in EMEM (Biowhittaker) supplemented with 10% FBS (Biowhittaker) and 1% L-glutamine. Forty-eight hours post-seeding, the cells were fixed with 4% (w/v) paraformaldehyde/PBS for 10 min at room temperature and 20 min at 37°C. After washing with PBS, cells were permeabilized with PBS containing 0.1% Triton X-100 for 10 min at room temperature and 20 min at 37°C. Cells were then incubated with monoclonal mouse anti-63 antibody in PBS +1% fetal bovine serum (FBS) for 1 h at 37°C. After washing with PBS and FBS (1%), coverslides were incubated with a Texas Red- conjugated anti-mouse secondary antibody (1/1000; Molecular Probes) for 1 h at 37°C. Following a water wash, cells were observed by fluorescent microscopy (Nikon).

### Western Blot analysis

HeLa or MeWo cells expressing or not wild-type or mutated IE63 were lysed in radioimmunoprecipitation assay buffer (10 mM Tris-HCl pH 8, 100 mM NaCl, 1 mM EDTA, 1% NP-40, 0.5% SDS) containing complete protease inhibitor cocktail (Roche), and 5 μg of proteins were separated on a 10% SDS-polyacrylamide gel. Proteins were transferred to nitrocellulose and detected with a mouse monoclonal anti-63 antibody. Western Blot analysis of IκBα degradation was done using a mouse monoclonal antibody directed against IκBα (kind gift from Ron Hay, Scotland) and a secondary horseradish peroxidase-conjugated rabbit anti-mouse antibody (Dako A/S). Western Blot using antibody directed against β-actin was used as loading control.

### Regulatory properties test

Transient transfection studies were carried out with HeLa cells (HeLa-IE63 and HeLa-Inv) seeded into 35-mm diameter 6-well cluster dishes using the FuGENE6 transfectant reagent (Roche). The plasmid pPol-luc, where the Firefly luciferase gene is under the control of the VZV DNA polymerase gene promoter [25], was used as reporter vector. The same quantity of each plasmid was transfected in each experiment. Twenty-four hours post-transfection, luciferase assays were performed using "Luciferase Reporter Gene Assay, High Sensitivity" kit (Roche) according to the manufacturer's instructions. For each experiment, the concentration of proteins was measured to normalize the results. Data from luciferase assays were collected from six independent transfection experiments. ρ-values were calculated using the graphpad quickcalcs software [59].

### Human gene micro-array analysis

Cells expressing or not wild-type or mutated IE63 were cultivated as described above then harvested and stored in RNA Later (Qiagen) at -70°C. Three independent experiments were performed, and the cell pellets were all processed in parallel. Total RNA was extracted from the cell pellets using the Rneasy mini kit (Qiagen). Synthesis of double-stranded DNA, *in vitro *transcription of biotin-labelled RNA, and fragmentation of labelled RNA were performed using the Genechip One-Cycle Target Labeling and Reagents kit (Affymetrix). The cRNA were then hybridized to U133A GeneChips (Affymetrix). The data generated from the three independent experiments were analyzed using the MSCL Analyst's Toolbox [60]. Affymetrix MAS5.0 signal values were retrieved, and a SSG transformation was applied. Only the significant differences based on ρ-values retrieved from three independent experiments were considered further. Comparisons were done using a one-way analysis of variance with transformed normalized data.

### Real-time PCR

Real-time PCR amplification was performed using 10 ng of each purified cDNA. The primers used were designed using the software Primers Express™ and were obtained from Eurogentec (Table 3).

**Table 3:** 

**Gene**	**Forward primer**	**Reverse primer**
**C3**	5'-gagccaccgaaaaatggaatc-3'	5'-gatccctttcttgtccgacatg -3'
**GATA3**	5'-tgaagcctaaacgcgatggata-3'	5'-ggtccagattcagtggttggaa-3'
**RCE1**	5'-tggagttgcccattttcacc-3'	5'-aagcagtgtaggcaccgaagac-3'
**RAB8B**	5'-cgccttcaacaccaccttcat-3'	5'-cgctgtgtcccatatctgaagc-3'
**WIF1**	5'-tggcagatccaaccgtcaat-3'	5'-tgccaccccatcctgtttt-3'
**CYFIP2**	5'-tccgagaggccaatcacaat-3'	5'-cgcacaaaacggttagtggac-3'
**NELL2**	5'-gcccagatcttaatcgcacct-3'	5'-gcttggctgatgttttggct-3'
**GPR37**	5'-gtcatgtgtctgtccgtggtga-3'	5'-ttggagatgctccgcatgtag-3'
**KCNJ12**	5'-tttctggtgtcgcccatca-3'	5'-ccaggatgaccacgatctcaa-3'
**HSP70**	5'-agcagacgcagatcttcacca-3'	5'-aagcgccccaacagattgt-3'
**ICAM1**	5'-agaccttagcgcggtgtaga-3'	5'-agtagcagaggagctcagcg-3'
**IL8**	5'-gggccatcagttgcaaatc-3'	5'-ttccttccggtggtttcttc-3'
**IL6**	5'-aagcacactttccccttcc-3'	5'-ctatcgttcttggtgggctc-3'
**IκBα**	5'-ccaaccagccagaaattgct-3'	5'-tctcggagctcaggatcaca-3'
**TRAF1**	5'-gctttttattgttcccacggct-3'	5'-actcgctaggccagaccttcat-3'
**HSPA6**	5'-ccattgacgctggtgtctttg-3'	5'-cgccggaattcttccatga-3'
**MCP1**	5'-tctcgcctccagcatgaaagt-3'	5'-gcattgattgcatctggctga-3'
**HLA-DRB1**	5'-cccagtactggaacagccagaa-3'	5'-tgcactgtgaagctctcaccaa-3'
**β2-microglobuline**	5'-gagtatgcctgccgtgtg-3'	5'-aatccaaatgcggcatct-3'

PCRs were run on an ABI 7700 instrument with the SYBR Green Master Mix (Applied Biosystems) and data were analyzed using Sequence Detector software (Applied Biosystems). Results were normalized using the β2-microglobulin transcripts. Experiments were done at least in triplicate. Differences (n-fold) between samples were calculated using the standard-curve method and the 2^-ΔCt ^method [61]. ρ-values were calculated using the graphpad quickcalcs software [59].

### Chromatin Immuno-precipitation assay

The ChIP assays were carried following the Upstate Cell Signalling protocol. In short, DNA was sonicated during twenty minutes (alternatively 30s of sonication followed by 30s of rest). The protein A Agarose beads (Pierce) were saturated with Herring Sperm DNA (Sigma-aldrich), 1 μg DNA/20 μL protein A Agarose. All ChIP assays were performed at least three times. qPCR targeting the promoter region of each gene were performed on the immunoprecipitated DNA. The primers used were designed using the software Primers Express™: ICAM-1-FW (5'-cccgattgctttagcttggaa-3') and ICAM-1-RV (5'-ccggaacaaatgctgcagttat-3'), IL-8-FW (5'-gccatcagttgcaaatcgtg-3') and IL-8-RV (5'-agtgctccggtggctttt-3'); IκBα-FW (5'-cgctcatcaaaaagttccctg-3') and IκBα-RV (5'-ggaatttccaagccagtcagac-3'). For unspecific binding to the beads, treated cells extracts were incubated with 2 μg of unspecific antibody (Flag, Sigma-aldrich). The following antibodies were used: p65, H3AcK9, HDAC3, Histone H3, and Human RNA polymerase II. Data were analysed using AbiPrism 7000 Sequence Detection System Software. Ct values obtained were normalized following Livak *et al*. [61]. ρ-values were calculated using the graphpad quickcalcs software [59].

## List of abbreviations

VZV, Varicella-Zoster Virus ; CDK1, Cyclin-Dependent Kinase 1 ; IE, Immediate Early ; E, Early ; L, Late ; IE63, Immediate Early 63 protein ; ORF, Open Reading Frame ; HSV, Herpes Simplex Virus ; NFκB, Nuclear Factor κB ; IL-6/8, Interleukin 6/8 ; ICAM-1, Intercellular Adhesion Molecule-1 ; TNFα, Tumor Necrosis Factor α; ChIP assay, Chromatin Immunoprecipitation assay ; HDAC, Histone Deacetylase.

## Authors' contributions

LH constructed the cell lines expressing IE63, carried out the micro-arrays analysis, western-blotting analysis and regulatory properties of IE63. NEM set-up the ChIP analysis. EDV constructed the pTRI-EF1a-ORF63-IRES-EGFP vectors. CSD involved in drafting the manuscript and revising it critically for important intellectual content. SB obtained several mutated forms of IE63. JP contributed to the conception and design of the experiments, involved in drafting the manuscript and revising it critically for important intellectual content; and gave the final approval of the version to be published.
